# Comparative analysis of financial toxicity between SARS-CoV-2 infection and common comorbidities

**DOI:** 10.1371/journal.pone.0309116

**Published:** 2024-08-15

**Authors:** Han Su, Hilaire J. Thompson, Karl Cristie Figuracion, Mayur Bipin Patel, Dale M. Needham

**Affiliations:** 1 School of Nursing, Vanderbilt University, Nashville, TN, United States of America; 2 School of Nursing, University of Washington, Seattle, WA, United States of America; 3 Department of Radiation Oncology, School of Medicine, University of Washington, Seattle, WA, United States of America; 4 Division of Trauma, Emergency General Surgery and Surgical Critical Care, Vanderbilt University Medical Center, Nashville, TN, United States of America; 5 Critical Illness, Brain Dysfunction and Survivorship Center, Vanderbilt University Medical, Nashville, TN, United States of America; 6 Department of Medicine, Division of Pulmonary and Critical Care Medicine, Johns Hopkins University School of Medicine, Baltimore, MD, United States of America; 7 Department of Physical Medicine and Rehabilitation, Johns Hopkins University School of Medicine, Baltimore, MD, United States of America; 8 Outcomes After Critical Illness and Surgery (OACIS) Group, Johns Hopkins University, Baltimore, MD, United States of America; 9 School of Nursing, Johns Hopkins University, Baltimore, MD, United States of America; Kyung Hee University School of Medicine, REPUBLIC OF KOREA

## Abstract

Financial toxicity is common in individuals with COVID-19 and Long COVID. However, the extent of financial toxicity experienced, in comparison to other common comorbidities, is uncertain. Contributing factors exacerbating financial challenges in Long COVID are also unclear. These knowledge gaps are addressed via a cross-sectional analysis utilizing data from the 2022 National Health Interview Survey (NHIS), a representative sample drawn from the United States. COVID-19 cases were identified through self-reported positive testing or physician diagnoses. Long COVID was defined as experiencing COVID-19-related symptoms for more than three months. Comorbidity was assessed based on self-reported diagnoses of ten doctor-diagnosed conditions (Yes/No). Financial toxicity was defined as having difficulty paying medical bills, cost-related medication nonadherence, delaying healthcare due to cost, and/or not obtained healthcare due to cost. A total of 27,492 NHIS 2022 respondents were included in our analysis, representing 253 million U.S. adults. In multivariable logistic regression models, adults with Long COVID (excluding respondents with COVID-19 but not Long COVID), showed increased financial toxicity compared to those with other comorbidities, such as epilepsy (OR [95% CI]: 1.69 [1.22, 2.33]), dementia (1.51 [1.01, 2.25]), cancer (1.43 [1.19, 1.71]) or respiratory/cardiovascular conditions (1.18 [1.00, 1.40]/1.23 [1.02, 1.47]). Long COVID-related financial toxicity was associated with female sex, age <65 years, lack of medical insurance, current paid employment, residence region, food insecurity, fatigue, mild to severe depression symptoms experienced during the survey completion, visits to hospital emergency rooms, presence of arthritis, cardiovascular or respiratory conditions, and social activity limitations. In conclusion, American adults with Long COVID, but not those who had prior COVID-19 infection without Long COVID, exhibited a higher prevalence of financial toxicity compared to individuals with common comorbidities. Vulnerable populations were at greater risk for financial toxicity. These findings emphasize the importance of evaluating strategies to reduce economic burden and increase awareness of the effect of Long COVID-related financial toxicity on patient’s healthcare and health status.

## Introduction

Globally, the COVID-19 pandemic has infected >770 million people [1], among whom >5–10% have developed persistent symptoms, such as fatigue, cognitive difficulties, and mood changes, collectively known as Long COVID [2–6]. Long COVID, lasting for months, has been associated with increased healthcare utilization, higher disability insurance claims, and reduced productivity [3, 7–16]. Long COVID imposes a significant burden of 80 disability-adjusted life years [DALYs] per 1,000 individuals among non-hospitalized adults and 643 DALYs per 1,000 individuals among hospitalized adults over a two-year period [11]. These rates surpass the burden attributed to either cancer (50 DALYs per 1,000 Americans) or heart disease (52 DALYs per 1,000 Americans] [17]. Consequently, these sequelae incur an estimated total cost exceeding $2.6 trillion in the US [18], alongside the potential negative repercussions of this economic strain on medical care, known as financial toxicity [19].

Evidence indicates that financial toxicity is prevalent among adults affected by COVID-19 and/or Long COVID, not only in the U.S. [3, 8, 20–22] but also globally[23–25]. it remains uncertain how this compares to the financial toxicity associated with other common comorbidities. Also, we lack understanding of factors associated with higher financial burden in Long COVID. This knowledge gap hampers policymaking for Long COVID care infrastructure and the design and evaluation of interventions to reduce related financial burdens.

Expanding on prior research [22], which utilized data from the 2022 U.S. National Health Interview Survey (NHIS), our study aims to examine the prevalence of financial toxicity among Long COVID patients and individuals who experienced COVID-19 only (without Long COVID), compared to those with common comorbidities. Furthermore, the study seeks to analyze factors correlated with increased financial burden due to Long COVID also using 2022 NHIS.

## Methods

### Data source

The NHIS, initiated in 1957 and conducted annually by the National Center for Health Statistics, employs geographically clustered sampling for nationally representative data collection from civilian, noninstitutionalized populations in all 50 US states and the District of Columbia. NHIS data assist the Department of Health and Human Services (HHS) in monitoring health trends and tracking national health goals. This survey covers demographics, health, behavior, disability, and social functioning. NHIS content undergoes periodic updates, with changes in 2022 specifically to include questions about Long COVID. Interviews typically occur in respondents’ homes, with 55.7% partially or entirely conducted by telephone in 2022 due to the COVID-19 pandemic [26, 27]. Under the U.S. Department of Health and Human Services (HHS) regulations for the protection of human subjects in research (45CFR 46), research involving publicly available data sets do not require IRB review.

### Study sample

Our analysis utilized data from the 2022 NHIS Sample Adult Core [28]. We included all participants who have a valid answer and exclude those with missing or unknown answers on COVID-19 questions. COVID-19 infection was identified by a ’yes’ response to survey questions: ’Has a doctor or other health professional ever told you that you had or likely had coronavirus or COVID-19?’ or ’Did you ever take a test that showed you had coronavirus or COVID-19?’ Long COVID was ascertained by a "yes" response to the survey question: ‘Did you experience symptoms lasting 3 months or more that were not present before having COVID-19?’ among those with mild to severe COVID-19 symptoms [27], aligning with the World Health Organization’s definition [29]. The covariates considered in this analysis, as outlined below, were selected based on prior research findings[2–16, 20–22], consistent with methodological recommendations for building regression models [30].

### Sociodemographic data

Sociodemographic variables included age, sex, race/ethnicity, education, marital status, insurance coverage, household income, and sources of income (categorical variable allowing for multiple simultaneous response options). Metropolitan residence was determined using the 2013 NCHS Urban-Rural classification scheme for counties, categorized as "yes" (large central, large fringe, medium, and small metro) or "no" (nonmetropolitan) [27, 31]. Food insecurity was assessed through 10 questions developed by the United States Department of Agriculture [32], with a score of 3–10 defining food insecurity [27, 33].

### Comorbidities and health service utilization

Comorbidity was determined based on self-report doctor-diagnosed conditions (yes/no): hypertension, high cholesterol, epilepsy, diabetes, respiratory conditions (asthma, COPD, emphysema, or chronic bronchitis), cardiovascular conditions (heart attack, coronary heart disease, angina, myocardial infarction, or stroke), arthritis, immunosuppression (weakened immune system due to prescriptions or health condition), dementia, and cancer.

Health service utilization includes yes/no responses regarding visits to urgent care centers or drug/grocery store clinics; hospital emergency room visits; overnight hospitalizations; receipt of physical, speech, or occupational therapy; home care; and counseling or therapy sessions with a mental health professional in the past 12 months.

### Social functioning, mental health, and fatigue

The NHIS assessed disability using the Washington Group Adult Composite Disability Indicator, developed by the World Health Organization (WHO). This measure has been validated and widely adopted as a standardized set of questions for identifying disability prevalence in population health surveys[34–36]. Adults reported their levels of function in vision, hearing, mobility, communication, cognition, and self-care. Individuals responding ’a lot of difficulty’ or ’cannot do at all’ in at least one of these domains were considered to have a disability [27, 37]. Social functioning was evaluated through two items related to independently completing errands and participation in social activities. Adults indicating "a lot of difficulty" or " cannot do at all " were classified as having an impairment in the corresponding item [27, 37].

Anxiety was evaluated using the 7-item Generalized Anxiety Disorder scale (GAD-7), which includes categories none or minimal (values ranging from 0 to 4) to mild to severe (values ranging from 5 to 21) based on their experiences in the past two weeks [38]. The reliability coefficient Cronbach’s α for the overall GAD-7 scale was 0.895 [39]. Depression was assessed using the 8-item Patient Health Questionnaire depression scale (PHQ-8), which was categorized into none or minimal (values ranging from 0 to 4) or mild to severe (values ranging from 5 to 24) based on their experiences in the past two weeks [40]. The Cronbach α for PHQ-8 was 0.82 [41]. Self-reported fatigue experienced during the past 3 months and self-reported moderate to severe COVID-19 symptoms (yes/no) were also collected.

### Primary outcome: Financial toxicity

Financial toxicity was defined based "yes/no" responses to the following items: difficulty paying medical bills, cost-related medication nonadherence (which includes skipping medication doses, taking less medication, delaying filling prescriptions to save money, or not obtaining prescription medication due to cost), delaying healthcare due to cost (referring to dental care, medical care, or counseling/therapy), and not obtained healthcare due to cost [22]. The metrics used in this study have shown robust correlations with objective financial toxicity resulting from out-of-pocket medical expenses in previous research [33, 42–44]. We calculated a composite score that represents the total number of financial toxicities, with scores ranging from 0 to 4. This approach has been used in another study [33]. Detailed content regarding all the questionnaires used in the study can be accessed at https://ftp.cdc.gov/pub/Health_Statistics/NCHS/Survey_Questionnaires/NHIS/2022/EnglishQuest-508.pdf

### Analyses

We summarized data using means and SDs for continuous variables, and proportions for categorical variables. We compared data using Student t tests for continuous variables, and chi square or Fisher exact tests, as appropriate, for categorical variables. All analyses utilized Stata version 17 (StataCorp, College Station, TX, USA), applying survey weights and the ’svy’ command to address the complex survey design of the NHIS and generate nationally representative estimates.[45] Statistical significance was indicated by a two-sided p-value <0.05.

To assess financial toxicity prevalence in adults who had Long COVID and who had COVID-19 only (without Long COVID), in comparison to other adults with other comorbidity, we utilized multivariable logistic regression and the ’lincom’ command with the NHIS 2022 dataset. The ’lincom’ command calculates point estimates, standard errors, and confidence intervals for linear combinations of coefficients subsequent to any estimation command, including survey estimation. The study’s binary outcome was whether individuals experienced any financial toxicity (Y/N). Exposure variables included SARS-CoV-2 infection status (Long COVID, COVID-19 only, and uninfected) and 10 comorbidities (yes/no), as described above. Covariates included measures of sociodemographic, health services utilization, disability, social functioning, mental health, and fatigue.

Finally, we utilized bivariable Poisson regression models and a multivariable Poisson model to identify factors associated with higher financial toxicity (indicating a greater total number of financial toxicities) in Long COVID cases. The comprehensive set of potential factors included sociodemographic factors, comorbidities, type of health service utilization, disability, social functioning, mental health, and fatigue [46, 47]. Multicollinearity was not present based on assessment using variance inflation factors (VIF), with a VIF greater than 10 indicating multicollinearity.

## Results

A total of 27,492 NHIS respondents were included in our analysis, representing 253 million U.S. adults. Table 1 presents the participants’ characteristics and financial toxicity of 2022 NHIS respondents. Of the 27,492 respondents, 78% were less than 65 years old, 62% were non-Hispanic white, 38% had a high school education or lower, 28% had family income below 200% of the federal poverty level, 10% were uninsured, and 8% experienced food insecurity. Notably, 8,334 (33%) had COVID-19 only, while 1,797 (7%) had Long COVID. Financial toxicity was reported by 32% of 2022 NHIS respondents. In the cohort of adults with long COVID, 47% reported experiencing financial toxicity. In contrast, among those with COVID-19 alone, 31% experienced financial toxicity. Regardless of the SARS-CoV-2 infection status (Long COVID, COVID-19 only, and uninfected), the three most commonly-reported types of financial toxicity were consistent: healthcare not obtained due to cost, delayed healthcare due to cost, and difficulty paying for medical bills (Table 1).

**Table 1:** Baseline characteristics and financial toxicity in the 2022 NHIS sample stratified by long COVID and SARS-CoV-2-infection status ^†^.

Variables	Total (n = 27,492)	With Long COVID (n = 1,797)	With COVID-19 only (n = 8,334)	No COVID-19 (n = 17,361)
**Weighted population size,** No. (%)	253,992,780 (100)	17,610,801 (7)	82,750,025 (33)	153,631,955(60)
**Male,** % (95% CI), weighted	49 (48, 49)	37 (34, 40)	50 (48, 51)	50 (49, 50)
**Age < 65 years,** % (95% CI), weighted	78 (77, 78)	87 (85, 88)	85 (84, 86)	73 (72, 74)
**Non-Hispanic White,** % (95% CI), weighted	62 (61, 64)	64 (61, 67)	61 (60, 63)	62 (61, 64)
**High School Graduate or lower,** % (95% CI), weighted	38 (37, 39)	34 (31, 37)	34 (33, 35)	40 (39, 41)
**Married or living with a partner,** % (95% CI), weighted	60 (60, 61)	64 (61, 66)	62 (61, 63)	59 (58, 61)
**Below 200% of the federal poverty level for family income,** % (95% CI), weighted.	28 (27, 29)	28 (25, 31)	24 (22, 25)	29 (28, 31)
**Currently work for pay,** % (95% CI), weighted.	61 (60, 62)	66 (63, 69)	70 (69, 71)	55 (54, 56)
**Source of income,** % (95% CI), weighted.				
Wages or salaries	81 (80, 81)	86 (84, 88)	87 (87, 88)	76 (76, 77)
Interest accounts, investments, or trusts	29 (28, 30)	23 (21, 25)	29 (27, 30)	30 (29, 31)
**Non-US Citizen,** % (95% CI), weighted	8.1 (7.5, 8.7)	6.4 (5.0, 8.1)	7.9 (7.1, 8.8)	8.4 (7.7, 9.2)
**Food insecurity** ^¶^	8.0 (7.5, 8.5)	13 (11, 15)	6.7 (6.1, 7.5)	8.1 (7.5, 8.7)
**Residence in metropolitan area** ^‡^, % (95% CI), weighted	86 (85, 87)	85 (82, 87)	87 (86, 88)	86 (85, 87)
**Residence region**				
Northeast	18 (17, 19)	17 (15, 20)	19 (17, 20)	17 (16, 18)
Midwest	21 (20, 22)	22 (20, 25)	21 (19, 22)	21 (19, 22)
South	38 (37, 40)	36 (33, 39)	38 (37, 40)	38 (37, 40)
West	24 (22, 25)	25 (22, 28)	22 (21, 24)	24 (23, 26)
**Insured,** % (95% CI), weighted	90 (90, 91)	91 (89, 92)	91 (90, 92)	90 (89, 90)
**Health service utilization,** % (95% CI), weighted				
Visited urgent care centers or drug/grocery store clinics	33 (32, 33)	44 (41, 47)	39 (37, 40)	28 (27, 29)
Visited hospital emergency room	20 (19, 21)	32 (30, 35)	20 (19, 21)	18 (18, 19)
Hospitalized overnight	8.4 (8.0, 8.8)	12 (11, 14)	8.5 (7.8, 9.2)	7.9 (7.4, 8.4)
Received counseling/therapy from mental health professional	13 (12, 13)	20 (17, 22)	13 (12, 14)	12 (11, 12)
Care at home from a nurse or other health professional	3.5 (3.3, 3.8)	4.3 (3.3, 5.6)	2.8 (2.5, 3.2)	3.8 (3.5, 4.1)
Received physical/speech/rehabilitative/occupational therapy	12 (11, 12)	15 (13, 17)	12 (11, 12)	12 (11, 12)
**Comorbidities,** ^‡‡^ % (95% CI), weighted				
Hypertension	32 (31, 33)	35 (33, 38)	28 (27, 29)	34 (33, 35)
High cholesterol	27 (27, 28)	28 (25, 30)	24 (23, 25)	29 (28, 30)
Diabetes	9.6 (9.2, 10)	11.0 (8.9, 12)	8.2 (7.6, 8.9)	10.0 (9.7, 11.0)
Arthritis	22 (21, 22)	27 (25, 29)	17 (16, 18)	23 (23, 24)
Dementia	1.1 (0.92, 1.2)	0.76 (0.42, 1.4)	0.62 (0.47, 0.81)	1.3 (1.1, 1.5)
Immunosuppression	7.4 (7.1, 7.8)	13 (11, 15)	7.1 (6.4, 7.8)	7.0 (6.5, 7.4)
Epilepsy	1.9 (1.7, 2.1)	2.2 (1.5, 3.2)	1.6 (1.3, 2.0)	2.0 (1.7, 2.2)
Cardiovascular conditions	8.5 (8.1, 8.8)	8.7 (7.3, 10.0)	6.3 (5.8, 6.9)	9.6 (9.1, 10.0)
Respiratory conditions	17 (17, 18)	27 (25, 30)	17 (16, 18)	16 (16, 17)
Cancer	9.6 (9.2, 9.9)	9.1 (7.8, 11.0)	7.4 (6.8, 8.0)	11.0 (10.0, 11.0)
**Disability** ^¦^^,^ % (95% CI), weighted	9.3 (8.8, 9.7)	13 (11, 15)	6.6 (6.0, 7.3)	10 (9.7, 11.0)
**Impaired Social Functioning,** % (95% CI), weighted				
Difficulty participating in social activities	4.4 (4.1, 4.7)	4.9 (3.9, 6.1)	3.2 (2.7, 3.7)	4.9 (4.6, 5.4)
Difficulty doing errands alone	4.5 (4.2, 4.8)	4.5 (3.5, 5.7)	3.0 (2.6, 3.5)	5.3 (4.9, 5.7)
**Experience fatigue in past 3 months,** % (95% CI), weighted	67 (66, 68)	83 (81, 85)	69 (68, 70)	64 (63, 65)
**Mild-Severe anxiety symptoms**^¢^^,^ % (95% CI), weighted.	18 (18, 19)	32 (30, 35)	18 (17, 19)	17 (16, 18)
**Mild-Severe depressive symptoms**^**&**^^,^ % (95% CI), weighted.	21 (21, 22)	37 (34, 39)	19 (18, 20)	21 (20, 21)
**Financial Toxicity,** % (95% CI), weighted				
Experience any financial toxicity ^§^	32 (31, 33)	47 (44, 50)	31 (30, 32)	31 (30, 31)
Difficulty Paying for medical bills.	11 (10, 11)	19 (17, 21)	10 (9.4, 11)	10 (9.5, 11)
Cost-related medication nonadherence *	7.1 (6.7, 7.4)	16 (14, 18)	6.3 (5.7, 7.0)	6.5 (6.0, 7.0)
Delayed healthcare due to cost ^$^	20 (19, 21)	31 (28, 34)	19 (18, 20)	19 (19, 20)
Not obtained healthcare due to cost ^!^	23 (23, 24)	35 (32, 38)	22 (21, 23)	22 (23, 23)
**Total number of financial toxicities,** ^€^ % (95% CI), weighted				
0	69 (68, 69)	54 (51, 56)	70 (68, 71)	70 (69, 71)
1	12 (12, 12)	15 (13,17)	12 (11, 13)	12 (11, 12)
2	12 (11, 12)	16 (14,18)	12 (11, 12)	12 (11, 12)
3	5 (5, 5.7)	10 (8, 12)	5 (4, 6)	5 (5, 6)
4	2 (2, 3)	6 (5, 7)	2 (2, 2)	2 (2, 2)

^†^ The sizes of populations of each group were estimated with the complex survey design and survey weights of the National Health Interview Survey. All presented percentages are weighted. All presented percentages are weighted.

^‡^ Assessed with the 2013 NCHS Urban-Rural classification scheme for counties, participants were categorized as "yes" (encompassing large central, large fringe, medium, and small metro areas) or "no" (nonmetropolitan).

^¶^ Assessed through a set of 10 questions sponsored by the United States Department of Agriculture, food security levels were defined as "food secure" for values ranging from 0 to 2 and "low to very low" for values ranging from 3 to 10.

^‡‡^ Immunosuppression, characterized by a weakened immune system due to prescriptions or underlying health conditions. Cardiovascular conditions, including heart attack, coronary heart disease, angina, myocardial infarction, or stroke. Respiratory conditions, including asthma, COPD, emphysema, or chronic bronchitis.

^¦^ Assessed using the Washington Group Adult Composite Disability Indicator across seeing, hearing, mobility, communication, cognition, and self-care domains. Individuals reporting ’a lot of difficulty’ or ’cannot do at all’ in at least one domain were considered to have a disability.

^¢^Assessed with the 7-item Generalized Anxiety Disorder scale, respondents were categorized into two groups at the time of completing the 2022 National Health Interview Survey (NHIS): none/minimal (0–4) and mild to severe (5–21).

^**&**^ Assessed with the 8-item Patient Health Questionnaire depression scale, respondents were categorized into two groups at the time of completing the 2022 National Health Interview Survey (NHIS): none/minimal (0–4) and mild to severe (5–24)

^§^ In the past 12 months, individuals have experienced difficulty paying for medical bills, cost-related medication nonadherence, delayed care due to cost, or not obtained healthcare due to cost.

*In the past 12 months, individuals skipped medication doses, took less medication, delayed filling prescriptions to save money, or needed prescription medication but did not obtain it due to cost.

^$^ In the past 12 months, dental care, medical care, or counseling/therapy has been delayed due to cost.

^!^ In the past 12 months, there has been a need for dental care, medical care, or counseling/therapy that wasn’t obtained due to cost.

^**€**^A sum was calculated based on whether the respondent had: difficulty paying for medical bills, cost-related medication nonadherence, delayed care due to cost, or not obtained healthcare due to cost. The range of the sum is from 0 to 4.

Abbreviations: 95% confidence interval = 95% CI

### Prevalence of financial toxicity by SARS-CoV-2 infection vs. other comorbidities

**Fig 1:**
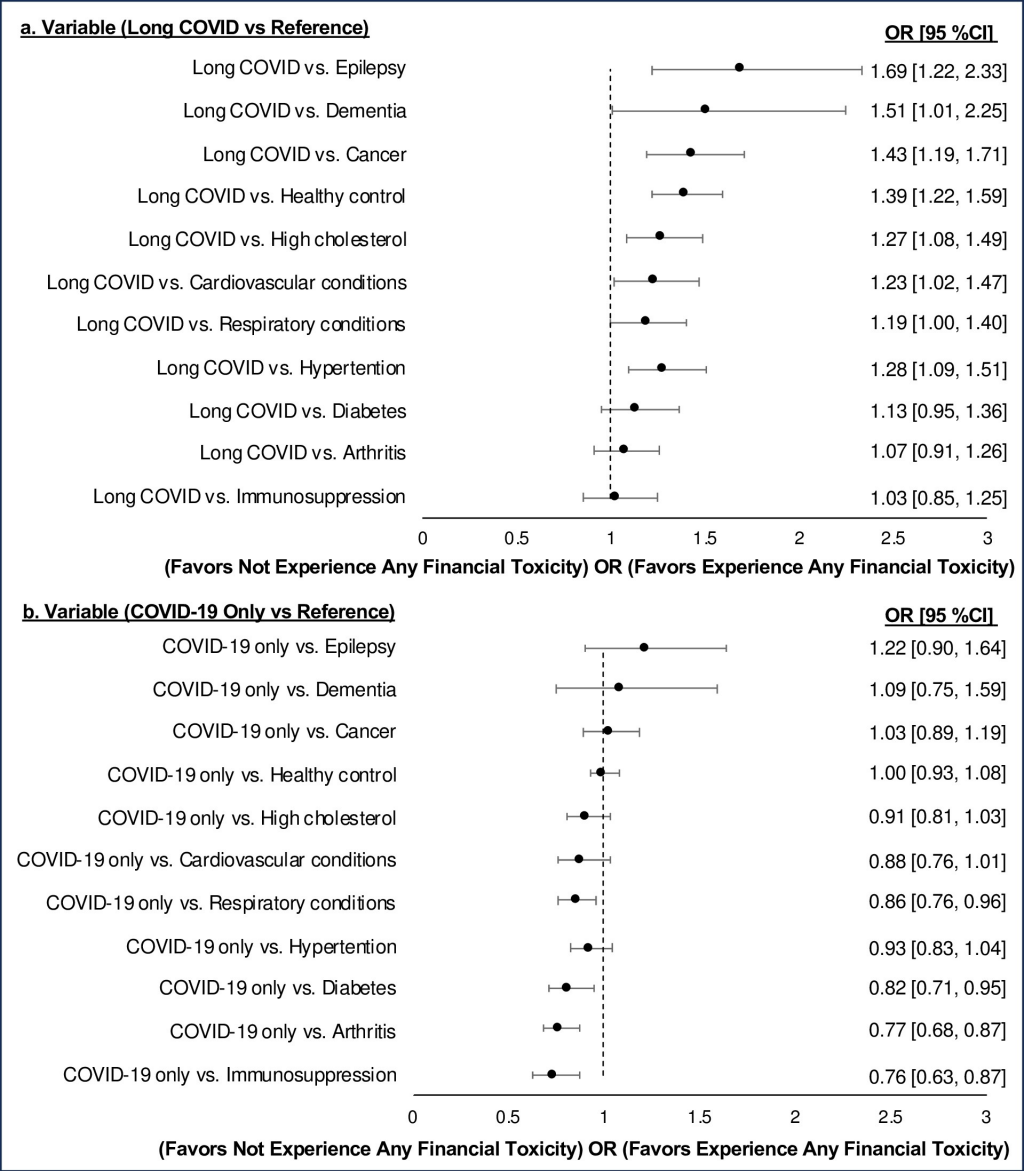
Odds Ratio for Experiencing Financial Toxicity in Adults with (a) Long COVID and (b) COVID-19 Only Compared to Uninfected Adults with Various Comorbidities in the National Health Interview Survey, 2022 Cohort (n = 25,056, weighted sample = 229,940,018) ^†^.

In a multivariable logistic regression analysis, adults with Long COVID exhibited higher odds of experiencing financial toxicity compared to both uninfected adults with various chronic diseases and healthy controls (uninfected individuals without any comorbidities). These included epilepsy (OR [95% CI]: 1.69 [1.22, 2.33], p = 0.002), dementia (1.51 [1.01, 2.25], p = 0.04), cancer (1.43 [1.19, 1.71], p<0.001), respiratory conditions (1.18 [1.00, 1.40], p = 0.05), high cholesterol (1.27 [1.08, 1.49], p = 0.004), cardiovascular conditions (1.23 [1.02, 1.47], p = 0.03), hypertension (1.28 [1.09, 1.51], p = 0.002), and health control (1.39 [1.21, 1.59], p<0.001) (Table 2 and Fig 1). In contrast, those who had COVID-19 alone did not show increased odds.

**Table 2:** Associations of risk factors for indicator for experiencing any financial toxicity in the national health interview survey, 2022 cohort (n = 25,056, weighted sample = 229,940,018) ^†^.

Variables	Bivariable logistic regression		Multivariable logistic regression	
	OR (95% CI)	p value	OR (95% CI)	p value
**SARS-CoV-2-infection Status**				
No COVID-19	Reference		Reference	
COVID-19 only	1.01 (0.94, 1.08)	0.81	1.00 (0.93, 1.08)	0.93
Long COVID	1.99 (1.78, 2.24)	<0.001	1.39 (1.22, 1.59)	<0.001
**Male**	0.76 (0.71, 0.81)	<0.001	0.84 (0.77, 0.91)	<0.001
**Age < 65 years**	1.76 (1.64, 1.89)	<0.001	1.30 (1.16, 1.46)	<0.001
**Non-Hispanic White**	0.66 (0.62, 0.71)	<0.001	0.95 (0.87, 1.04)	0.31
**High School Graduate or lower**	1.53 (1.44, 1.64)	<0.001	1.07(0.99, 1.16)	0.10
**Married or living with partner**	0.76 (0.71, 0.81)	<0.001	0.96 (0.89, 1.03)	0.28
**Below 200% of the federal poverty level for family income**	2.37 (2.21, 2.55)	<0.001	1.45 (1.32, 1.59)	<0.001
**Currently work for pay**	0.99 (0.93, 1.06)	0.86	1.11 (1.01, 1.24)	0.04
**Source of income**				
Wages or salaries	1.16 (1.07, 1.25)	<0.001	1.20 (1.06, 1.35)	0.003
Interest accounts, investments, or trusts	0.42 (0.39, 0.46)	<0.001	0.64 (0.58, 0.69)	<0.001
**Non-US citizenship**	1.91 (1.69, 2.16)	<0.001	1.30 (1.11, 1.51)	0.001
**Food Insecurity** ^¶^	5.41 (4.77, 6.13)	<0.001	2.67 (2.33, 3.06)	<0.001
**Residence in metropolitan area** ^‡^	0.99 (0.89, 1.10)	0.79	1.09 (0.95, 1.24)	0.22
**Residence Region**				
Northeast	Reference		Reference	
Midwest	1.15 (1.02, 1.28)	0.02	1.08 (0.94, 1.24)	0.27
South	1.51 (1.36, 1.68)	<0.001	1.31 (1.16, 1.49)	<0.001
West	1.33 (1.18, 1.50)	<0.001	1.27 (1.11, 1.46)	<0.001
**Insured**	0.25 (0.23, 0.28)	<0.001	0.28 (0.25, 0.32)	<0.001
**Health service utilization**				
Visited urgent care centers or drug/grocery store clinics	1.23 (1.15, 1.31)	<0.001	1.12 (1.03, 1.21)	0.008
Visited hospital emergency room	2.00 (1.86, 2.15)	<0.001	1.36 (1.24, 1.49)	<0.001
Hospitalized overnight	1.61 (1.46, 1.78)	<0.001	1.16 (1.02, 1.32)	0.03
Received counseling/therapy from mental health professional	2.02 (1.86, 2.20)	<0.001	1.30 (1.17, 1.46)	<0.001
Care at home from a nurse or other health professional	1.02 (0.87, 1.20)	0.77	0.68 (0.55, 0.85)	0.001
Received physical/speech/rehabilitative/occupational therapy	1.10 (1.00. 1.20)	0.04	1.04 (0.93, 1.17)	0.49
**Comorbidities** ^‡‡^				
Hypertension	1.11 (1.04, 1.19)	0.002	1.08 (0.99, 1.18)	0.08
High cholesterol	1.04 (0.97, 1.11)	0.24	1.10 (1.01, 1.20)	0.04
Diabetes	1.38 (1.26, 1.51)	<0.001	1.23 (1.09, 1.38)	0.001
Arthritis	1.31 (1.22, 1.41)	<0.001	1.30 (1.18, 1.43)	<0.001
Dementia	1.02 (0.78, 1.35)	0.86	0.92 (0.63, 1.34)	0.67
Immunosuppression	1.75 (1.57, 1.94)	<0.001	1.35 (1.18, 1.55)	<0.001
Epilepsy	1.40 (1.13, 1.73)	0.002	0.82 (0.62, 1.10)	0.19
Cardiovascular conditions	1.26 (1.14, 1.40)	<0.001	1.13 (0.999, 1.28)	0.051
Respiratory conditions	1.57 (1.45, 1.69)	<0.001	1.17(1.07, 1.29)	0.001
Cancer	0.80 (0.72, 0.88)	<0.001	0.97 (0.86, 1.11)	0.67
**Disability** ^¦^	1.88 (1.70, 2.07)	<0.001	1.07 (0.92, 1.24)	0.38
**Impaired Social Functioning**				
Difficulty doing errands alone	1.44 (1.26, 1.64)	<0.001	0.87 (0.69, 1.10)	0.25
Difficulty participating in social activities	1.88 (1.64, 2.15)	<0.001	0.93 (0.74, 1.17)	0.54
**Experience fatigue in past 3 months**	2.00 (1.87, 2.14)	<0.001	1.40 (1.29, 1.51)	<0.001
**Mild -Severe anxiety symptoms** ^**¢**^	3.22 (2.98, 3.49)	<0.001	1.66 (1.49, 1.86)	<0.001
**Mild -Severe depressive symptoms** ^**&**^	3.15 (2.92, 3.39)	<0.001	1.62 (1.45, 1.80)	<0.001

^†^ The sizes of populations of each group were estimated with the complex survey design and survey weights of the National Health Interview Survey. All presented percentages are weighted. All presented percentages are weighted.

^‡^ Assessed with the 2013 NCHS Urban-Rural classification scheme for counties, participants were categorized as "yes" (encompassing large central, large fringe, medium, and small metro areas) or "no" (nonmetropolitan).

^¶^ Assessed through a set of 10 questions sponsored by the United States Department of Agriculture, food security levels were defined as "food secure" for values ranging from 0 to 2 and "low to very low" for values ranging from 3 to 10.

^‡‡^ Immunosuppression, characterized by a weakened immune system due to prescriptions or underlying health conditions. Cardiovascular conditions, including heart attack, coronary heart disease, angina, myocardial infarction, or stroke. Respiratory conditions, including asthma, COPD, emphysema, or chronic bronchitis.

^¦^ Assessed using the Washington Group Adult Composite Disability Indicator across seeing, hearing, mobility, communication, cognition, and self-care domains. Individuals reporting ’a lot of difficulty’ or ’cannot do at all’ in at least one domain were considered to have a disability.

^¢^Assessed with the 7-item Generalized Anxiety Disorder scale, respondents were categorized into two groups at the time of completing the 2022 National Health Interview Survey (NHIS): none/minimal (0–4) and mild to severe (5–21).

^**&**^ Assessed with the 8-item Patient Health Questionnaire depression scale, respondents were categorized into two groups at the time of completing the 2022 National Health Interview Survey (NHIS): none/minimal (0–4) and mild to severe (5–24)

^§^ In the past 12 months, individuals have experienced difficulty paying for medical bills, cost-related medication nonadherence, delayed care due to cost, or not obtained healthcare due to cost.

*In the past 12 months, individuals skipped medication doses, took less medication, delayed filling prescriptions to save money, or needed prescription medication but did not obtain it due to cost.

^$^ In the past 12 months, dental care, medical care, or counseling/therapy has been delayed due to cost.

^!^ In the past 12 months, there has been a need for dental care, medical care, or counseling/therapy that wasn’t obtained due to cost.

^**€**^A sum was calculated based on whether the respondent had: difficulty paying for medical bills, cost-related medication nonadherence, delayed care due to cost, or not obtained healthcare due to cost. The range of the sum is from 0 to 4.

Abbreviations: 95% confidence interval = 95% CI; Odds Ratio = OR

^**†**^ The odds ratio was estimated through multivariable logistic regression, as presented in Table 2 using the ’lincom’ command. In each disease model, the reference group was set as the specific disease, with Long COVID or COVID-19 only serving as the comparison group. COVID-19 cases were identified via self-report positive testing or physician diagnoses, while Long COVID involves COVID-19-related symptoms persisting for more than 3 months.

Respiratory conditions, including asthma, COPD, emphysema, or chronic bronchitis, and cardiovascular conditions, such as heart attack, coronary heart disease, angina, myocardial infarction, or stroke, were considered. Immunosuppression, characterized by a weakened immune system due to prescriptions or underlying health conditions, was also included.

Healthy controls are defined as those uninfected individuals without any comorbidities.

Each disease model was adjusted for various demographic and health-related factors, including sex, age, race/ethnicity, education, marital status, family income, insurance coverage, and other 9 comorbidities. Additional adjustments were made for citizenship, residence in metropolitan areas, residence region, income sources, health service utilization, disability, social functioning, mental health, and fatigue. Analytic weights were applied to account for the complex sampling design.

In the graphical representation, each circle symbolizes one estimate, with the center indicating the point estimate (odds ratio) of the effect size, and the horizontal lines indicating the confidence interval around that estimate.

Abbreviations: Odds Ratio = OR, 95% Confidence Interval = 95% CI.

### Associated factors for greater financial toxicities in long COVID

Several factors were positively associated with financial toxicity indicators among individuals with Long COVID. These factors include female sex, age under 65 years, lacking insurance, currently employed, residence region, experiencing food insecurity, fatigue, and exhibiting mild to severe symptoms of depression. Additionally, individuals who visited hospital emergency rooms, had arthritis, cardiovascular or respiratory conditions, and experienced difficulty participating in social activities reported a greater number of financial toxicities. Conversely, individuals with Long COVID who relied on primary income from investments experienced fewer financial toxicities compared to those who did not (Table 3).

**Table 3:** Bivariable and multivariable associations of risk factors for number of financial toxicity indicators in individuals with long COVID from the national health interview survey, 2022 (n = 1774, weighted sample = 17,295,036) ^†^.

Variables	Bivariable Poisson regression		Multivariable Poisson regression	
	IRR (95% CI)	p value	IRR (95% CI)	p value
**Male**	0.69 (0.59, 0.81)	<0.001	0.81 (0.70, 0.93)	0.004
**Age < 65 years**	1.52 (1.25, 1.84)	<0.001	1.31 (1.04, 1.67)	0.03
**Non-Hispanic White**	0.79 (0.69, 0.91)	0.001	0.98 (0.84, 1.15)	0.82
**High School Graduate or lower**	1.33 (1.16, 1.52)	<0.001	1.11 (0.95, 1.29)	0.18
**Married or living with a partner**	0.74 (0.64, 0.86)	<0.001	0.94 (0.81, 1.09)	0.43
**Below 200% of the federal poverty level for family income**	1.77 (1.53, 2.04)	<0.001	1.05 (0.89, 1.24)	0.53
**Currently work for pay**	0.87 (0.75, 1.02)	0.08	1.21 (1.03, 1.43)	0.02
**Source of income**				
Wages or salaries	0.94 (0.77, 1.14)	0.51	1.07 (0.86, 1.34)	0.53
Interest accounts, investments, or trusts	0.65 (0.54, 0.79)	<0.001	0.82 (0.70, 0.97)	0.02
**Non-US Citizen**	1.61 (1.29, 2.00)	<0.001	1.14 (0.90, 1.44)	0.29
**Food insecurity** ^¶^	2.66 (2.33, 3.05)	<0.001	1.66 (1.42, 1.93)	<0.001
**Residence in metropolitan area** ^‡^	1.08 (0.90, 1.31)	0.41	1.18 (0.98, 1.43)	0.08
**Residence region**				
Northeast	Reference		Reference	
Midwest	1.07 (0.82, 1.40)	0.63	1.06 (0.82, 1.37)	0.66
South	1.25 (0.98, 1.60)	0.07	1.26 (1.00, 1.58)	0.05
West	1.29 (1.00, 1.66)	0.05	1.27 (0.99, 1.62)	0.053
**Insured**	0.48 (0.41, 0.57)	<0.001	0.52 (0.43, 0.64)	0.00
**Health service utilization**				
Visited urgent care centers or drug/grocery store clinics	1.20 (1.04, 1.38)	0.01	1.10 (0.96, 1.25)	0.18
Visited hospital emergency room	1.74 (1.51, 2.00)	<0.001	1.24 (1.07, 1.43)	0.004
Hospitalized overnight.	1.58 (1.33, 1.87)	<0.001	1.11 (0.91, 1.36)	0.28
Received counseling/therapy from mental health professional	1.55 (1.34, 1.79)	<0.001	1.02 (0.87, 1.19)	0.82
Care at home from a nurse or other health professional	1.52 (1.16, 2.00)	0.003	1.07 (0.76, 1.49)	0.71
Received physical/speech/rehabilitative/occupational therapy	1.21(0.99, 1.47)	0.06	0.92 (0.75, 1.12)	0.39
**Comorbidities** ^‡‡^				
Hypertension	1.12 (0.97, 1.30)	0.11	1.05 (0.90, 1.22)	0.55
High cholesterol	1.10 (0.94, 1.28)	0.23	1.09 (0.93, 1.28)	0.29
Diabetes	1.32 (1.08, 1.61)	0.007	1.03 (0.84, 1.26)	0.80
Arthritis	1.46 (1.28, 1.67)	<0.001	1.19 (1.03, 1.38)	0.02
Dementia	1.68 (1.00, 2.81)	0.05	0.93 (0.62, 1.39)	0.71
Immunosuppression	1.62 (1.36, 1.92)	<0.001	1.05 (0.89, 1.25)	0.55
Epilepsy	1.44 (1.06, 1.96)	0.02	0.84 (0.57, 1.24)	0.37
Cardiovascular conditions	1.52 (1.27, 1.82)	<0.001	1.41 (1.16, 1.69)	<0.001
Respiratory conditions	1.51 (1.31, 1.73)	<0.001	1.20 (1.03, 1.39)	0.02
Cancer	1.21 (0.98, 1.49)	0.08	1.16 (0.95, 1.41)	0.16
**Disability** ^¦^	1.80 (1.53, 2.10)	<0.001	1.05 (0.87, 1.28)	0.60
**Impaired Social Functioning**				
Difficulty participating in social activities	2.04 (1.69, 2.47)	<0.001	1.40 (1.08, 1.83)	0.01
Difficulty doing errands alone	1.98 (1.60, 2.47)	<0.001	0.90 (0.67, 1.2)	0.47
**Experience fatigue in past 3 months**	1.97 (1.53, 2.53)	<0.001	1.38 (1.08, 1.76)	0.01
**Mild-Severe anxiety symptoms**	1.93 (1.68, 2.21)	<0.001	1.12 (0.94, 1.33)	0.20
**Mild-Severe depressive symptoms** ^**&**^	2.18 (1.88, 2.53)	<0.001	1.50 (1.25, 1.81)	<0.001
**Experience moderate to severe COVID-19 symptoms at their worst during acute infection**	1.35 (1.11, 1.63)	0.003	1.18 (0.99, 1.39)	0.06

^†^ The sizes of populations of each group were estimated with the complex survey design and survey weights of the National Health Interview Survey. All presented percentages are weighted. All presented percentages are weighted.

^‡^ Assessed with the 2013 NCHS Urban-Rural classification scheme for counties, participants were categorized as "yes" (encompassing large central, large fringe, medium, and small metro areas) or "no" (nonmetropolitan).

^¶^ Assessed through a set of 10 questions sponsored by the United States Department of Agriculture, food security levels were defined as "food secure" for values ranging from 0 to 2 and "low to very low" for values ranging from 3 to 10.

^‡‡^ Immunosuppression, characterized by a weakened immune system due to prescriptions or underlying health conditions. Cardiovascular conditions, including heart attack, coronary heart disease, angina, myocardial infarction, or stroke. Respiratory conditions, including asthma, COPD, emphysema, or chronic bronchitis.

^¦^ Assessed using the Washington Group Adult Composite Disability Indicator across seeing, hearing, mobility, communication, cognition, and self-care domains. Individuals reporting ’a lot of difficulty’ or ’cannot do at all’ in at least one domain were considered to have a disability.

^¢^Assessed with the 7-item Generalized Anxiety Disorder scale, respondents were categorized into two groups at the time of completing the 2022 National Health Interview Survey (NHIS): none/minimal (0–4) and mild to severe (5–21).

^**&**^ Assessed with the 8-item Patient Health Questionnaire depression scale, respondents were categorized into two groups at the time of completing the 2022 National Health Interview Survey (NHIS): none/minimal (0–4) and mild to severe (5–24)

Abbreviation: 95% confidence interval = 95% CI, Incidence Rate Ratio = IRR

## Discussion

Analyzing the 2022 U.S. NHIS, adults with Long COVID demonstrated a higher prevalence of financial toxicity indicators compared to individuals with various comorbidities (e.g., epilepsy, dementia, respiratory or cardiovascular conditions, cancer). This observation persisted even after adjusting for social demographic factors, healthcare utilization, and functional status. This difference was especially evident in specific toxicity measures, including not obtaining healthcare due to cost, delayed healthcare due to cost, and difficulty paying for medical bills. Factors associated with a higher total number of financial toxicity indicators in Long COVID included female sex, age <65 years, lack of medical insurance, current paid employment, food insecurity, fatigue, mild to severe depression symptoms experienced during the survey completion, visits to hospital emergency rooms, presence of arthritis, cardiovascular or respiratory conditions, and social activity limitations.

Our study demonstrates a significant prevalence of financial toxicity among adults with Long COVID, aligning with findings from previous U.S.-based studies [3, 8, 20–22] as well as international research [23–25]. A potential explanation is that chronic inflammation or other physiological processes causing prolonged symptoms, disability, or an elevated risk of developing cardiovascular disease [48], diabetes [49], neuropsychiatric sequelae [50], and acute or post-acute respiratory sequelae[51] could result in diminished work capacity [52], increased healthcare needs [53], and thereby contribute to financial burden. Further study is warranted to better understand the mechanisms underlying these effects. Additionally, our study contributes important new insights into this phenomenon. Firstly, this is the first study directly comparing financial toxicity among adults with Long COVID and who had COVID-19 only to adults with various comorbidities. Our results highlight those individuals with Long COVID, but not those who had COVID-19 only, face a greater financial burden than those with other chronic diseases, underscoring the significant impact of Long COVID-related financial toxicity. Second, financial toxicity encompasses both objective and subjective domains. Understanding subjective financial toxicity is crucial for evaluating how financial challenges affect healthcare delivery and the potential for seeking care [54]. Building on existing knowledge, we highlight the subjective domains, finding that the Long COVID-related financial burden adversely affects health-seeking behavior and medication adherence, potentially worsening health conditions and increasing morbidity and mortality. Third, our study identifies multifaceted factors associated with increased financial toxicity in adults with Long COVID, particularly among vulnerable populations, including individuals with low socioeconomic status, food insecurity, comorbidities, and no insurance. This underscores the need for implementing multilevel interventions involving patients, healthcare providers, as well as insurance and governmental-level initiatives to address Long COVID-related financial toxicity.

Currently, interventions involving both patients and healthcare providers have been developed to reduce the financial burden associated with medical care across a broad spectrum of disease populations [55, 56]. These interventions include screening individuals for medical-related financial hardships and social risk factors, enhancing discussions between patients and providers regarding healthcare costs, and providing financial education, counseling, and navigation services [55]. These interventions, shown to enhance patient financial literacy, alleviate anxiety related to healthcare costs, and improve treatment adherence [55], could be adapted for Long COVID. However, these interventions alone will not eliminate out-of-pocket expenses and associated perceived financial burden [55]. Therefore, additional efforts to evaluate methods to reduce financial toxicity are necessary.

During the early pandemic, the US needed to address issues related to the affordability of COVID-19 testing, vaccines, and treatment [57]. Government initiatives, including the Families First Coronavirus Response Act (FFCRA) and the Coronavirus Aid, Relief, and Economic Security Act (CARES), aimed to alleviate financial burdens [57, 58]. Despite these efforts, survivors with COVID-19 and Long COVID continue to face financial challenges [3, 9, 11, 16, 22, 59], emphasizing the need for ongoing legislative efforts to address out-of-pocket expenses. Additionally, large-scale healthcare reforms for universal access to affordable care could better meet individual healthcare needs and equip the nation for future health crises [60]. For working-age individuals with Long COVID, facilitating employment opportunities is crucial for financial stability and continued access to healthcare insurance [18]. Such work accommodations may include flexible work schedules and telework support to help reduce Long COVID-related delays in return to employment.

Previous research has demonstrated that, compared to uninfected controls, survivors of acute COVID-19 experience a substantial 12-month disease burden, with hazard ratios (HR) per 1,000 persons for cardiovascular disease and diabetes reported as 45.29 [42.22, 48.45] and 13.46 [12.11, 14.84], respectively[48, 49]. Our study highlights that the burden of cardiovascular disease in Long COVID survivors is associated with an increased likelihood of experiencing financial toxicity, a relationship not observed for diabetes. Future research should explore the mechanisms linking disease burden, particularly cardiovascular disease, to financial toxicity to develop strategies to mitigate these effects. Interestingly, our study indicates that individuals with Long COVID residing in the Midwest or Southern regions of the United States are more likely to experience a greater number of financial toxicities compared to those living in the Northeast. This disparity may be attributed to the distinct economic activities and environmental characteristics inherent to each region, which can influence financial burdens. Future research should investigate the specific factors contributing to these regional differences in financial toxicity.

Our study has notable strengths, including a comprehensive analysis of the prevalence and magnitude of financial toxicity in Long COVID, utilizing a nationally representative cohort. By comparing Long COVID patients with those with other diseases, we highlight the distinct burden of Long COVID-related financial toxicity. Furthermore, we identify factors associated with Long COVID-related financial toxicity, providing valuable insights for interventions and policy development. Potential limitations include potential bias and measurement error through self-reporting. However, the NHIS is a nationally recognized survey administered by the U.S. government, with NHIS employing rigorous techniques enhance response rates and ensure data quality. This NHIS methodology fosters greater candor and reduces potential biases associated with socially desirable responses, while also helping to address respondent fatigue, impatience, and accommodating individuals with cognitive and hearing impairments [61]. In order to mitigate biases arising from coverage, nonresponse, and sampling variability, NHIS weighting procedures systematically incorporate adjustments to align estimates with US Census Bureau population data. This process includes harmonizing estimates across demographics such as age, sex, race, ethnicity, educational attainment, and subnational geographical factors such as census division and metropolitan statistical area classification [62].

Moreover, the cross-sectional nature of the survey prevents us from establishing causality. Although it is not possible to randomize someone to having vs. not having Long COVID, further causal inference studies are required to help ascertain whether financial toxicity is caused by Long COVID. Despite our adjustments for potential confounders, residual confounding may remain. Factors such as the severity of comorbid conditions, the type and duration of Long COVID symptoms [63], and variability in COVID-19 treatments—such as vaccination [64] and administration of antiviral treatments [65].

In addition, although we examined the financial burden from various perspectives, our approach may not fully capture its entirety. Specifically, it may not account for instances of declared bankruptcy, depletion of savings, or the inability to pay for necessities. Future studies could explore the incorporation of qualitative data to achieve a more nuanced understanding. Lastly, adults in the US incur greater out-of-pocket healthcare expenses than other countries that may limit the international generalizability of these findings.

## Conclusion

Adults with Long COVID were more likely to experience financial toxicity compared to individuals with various comorbidities (e.g., epilepsy, dementia, respiratory or cardiovascular conditions, cancer). This finding was specific to Long COVID in those adults with COVID only (i.e., SARS CoV2 infection without Long COVID) did not have increased financial toxicity. Factors associated with increased financial toxicity in Long COVID included markers of social vulnerability, such as lower income, lack of healthcare insurance, and food insecurity. This finding emphasizes the need to explore and evaluate strategies to reduce the economic burden and improve healthcare for adults with Long COVID in order to maximize long-term health and well-being.
